# The Long and Winding Road for Symbiont and Yolk Protein to Host Oocyte

**DOI:** 10.1128/mBio.02997-20

**Published:** 2021-02-09

**Authors:** Takema Fukatsu

**Affiliations:** a Bioproduction Research Institute, National Institute of Advanced Industrial Science and Technology (AIST), Tsukuba, Japan; b Department of Biological Sciences, Graduate School of Science, The University of Tokyo, Tokyo, Japan; c Graduate School of Life and Environmental Sciences, University of Tsukuba, Tsukuba, Japan

**Keywords:** symbiosis, *Nephotottix*, leafhopper, *Nasuia*, *Sulcia*, vitellogenin, porin, endocytosis, vertical transmission, oocyte

## Abstract

Many insects are intimately associated with microbial symbionts, which are passed to developing oocytes in the maternal body for ensuring vertical transmission to the next generation. Previous studies uncovered that some symbionts utilize preexisting host’s molecular and cellular machineries for targeting oocytes. For example, the major yolk protein vitellogenin (Vg) is massively produced in fat body cells, processed and transported to ovaries, and incorporated into developing oocytes via Vg receptor (VgR)-mediated endocytosis, and some symbiotic bacteria were reported to interact with Vg and migrate to oocytes by hitchhiking the VgR-mediated endocytotic mechanism. In a recent study, Mao et al. (mBio 12:e01142-20, 2020, https://doi.org/10.1128/mBio.01142-20) reported that, in some leafhoppers, a considerable proportion of Vg is incorporated into symbiotic bacteria and translocated into oocytes by hitchhiking the symbiont’s vertical transmission mechanism, uncovering the host’s cooption of the symbiont’s oocyte-targeting machineries and highlighting complicated trajectories toward host-symbiont coevolution and integration.

## Abstract (precis)

Many insects are intimately associated with microbial symbionts, which are passed to developing oocytes in the maternal body for ensuring vertical transmission to the next generation. Previous studies uncovered that some symbionts utilize preexisting host’s molecular and cellular machineries for targeting oocytes.

## COMMENTARY

Many insects are either obligatorily or facultatively associated with microbial symbionts, which affect growth, survival, and reproduction of their hosts in a variety of ways. In most cases, such symbionts are maintained in somatic cells and organs of their hosts and are transovarially passed to developing oocytes for ensuring vertical transmission to the next generation (1, 2). The continuous vertical transmission of the symbiont through host generations over evolutionary time facilitates the loss of genes that are not needed for the intrahost life, the depletion of horizontally acquired genes from environmental sources, and the accumulation of deleterious mutations due to strong population bottlenecks, which tend to end up with symbiont genome reduction and uncultivability, thereby skewing the evolutionary trajectories of the symbiotic associations in an idiosyncratic manner (3, 4).

Apparently, the intrahost microbial journey is not an easy task, where the symbiont escapes from the original host cells, manages to pass through structural barriers, targets host’s ovarial tissue, and finally gets entry into host’s oocyte. Accomplishing this must require elaborate molecular and cellular mechanisms, which are likely preexisting as host’s molecular machineries to be utilized by the symbiont. Previous studies have shown that a variety of symbionts target host’s specific cellular components, like ovarial epithelia, germaria, nurse cells, follicle cells, germ/somatic stem cells, etc. (1, 5–8), by coopting the host’s molecular and cellular machineries, such as cytoskeletons, motor proteins, exo/endocytotic pathways, yolk transport systems, etc. (9–14).

Oogenesis requires much nutritional investment in the form of yolk, whose main component is the major yolk protein, called vitellogenin (Vg). Vg is synthesized in fat body cells, processed into mature forms, secreted to hemolymph, transported into growing oocytes via Vg receptor (VgR)-mediated endocytosis, and accumulated in the yolk granules in the storage form called vitellin (15). In *Spiroplasma*-infected fruit flies and *Wolbachia*-infected planthoppers, it was reported that the symbiotic bacteria are associated with Vg and transported into developing oocytes via the VgR-mediated yolk transport mechanism (12, 14). On account of the considerable size of bacterial cells, it is conceivable, though speculative, that the endocytosis-mediated Vg transport system is suitable for evolutionary cooption by the symbiotic bacteria for oocyte targeting and infection.

In this context, a totally unexpected phenomenon was recently reported by Mao et al. (16) in the rice green leafhopper *Nephotettix cincticeps.* The insect is obligatorily associated with dual genome-reduced endosymbiotic bacteria, *Sulcia* and *Nasuia*, in paired bacteriomes in the abdomen (17). *Sulcia* and *Nasuia* jointly synthesize essential amino acids for their leafhopper hosts and have cospeciated with them over evolutionary time, with *Sulcia* being more ancient than *Nasuia* (18). Using thorough histological, biochemical, and molecular genetic approaches, Mao et al. (16) demonstrated that (i) both *Sulcia* and *Nasuia* enter the posterior region of developing oocytes at the vitellogenic stage, when Vg is actively taken up by the germarium and then transported to the oocyte via a nutritive cord, (ii) in addition to the Vg localization spanning from germarium through nutritive cord to ooplasm, a considerable amount of Vg is detected in the posterior region of the oocytes, where Vg signals colocalize with *Nasuia* cells, (iii) confocal imaging and immunoelectron microscopy confirm that, strikingly, Vg is present inside the oocyte-invading *Nasuia* cells, (iv) RNA interference knockdown of VgR does not affect invasion of *Nasuia*-Vg complex into oocytes, uncovering that the *Nasuia*-associated Vg incorporation into oocytes is independent of the conventional VgR-mediated Vg transport mechanism, (v) yeast two-hybrid assay and glutathione *S*-transferase pulldown assay showed interaction between Vg and *Nasuia*’s porin, the major bacterial outer membrane channel protein, (vi) experimental suppression of *Nasuia* infection to oocytes results in reduced ovarial Vg, (vii) blocking of *Nasuia*’s porin by antibody injection causes reduced Vg titer in the oocytes and suppressed egg development and hatching, (viii) the *Nasuia*-Vg colocalization is also observed in other leafhopper species, and (ix) based on these results, it is proposed that the porin-mediated incorporation of Vg into the oocyte-infecting *Nasuia* cells constitutes another Vg transport system in addition to the conventional VgR-mediated Vg transport system in the leafhoppers.

Mao et al.’s (16) finding is particularly intriguing in the following respects. First, this study uncovers a previously unrecognized route of Vg transport to oocytes in insect development. Second, while previous studies identified symbiont’s cooption and utilization of host’s Vg transport mechanism for reaching oocytes (12, 14), this study suggests that, in contrast, the host side has coopted the symbiont’s oocyte targeting mechanism for Vg delivery to ooplasm. Here, the host’s pathway for Vg delivery for oocyte maturation and the symbiont’s pathway for vertical transmission to developing oocytes are intertwined in an evolutionary perspective. Considering that the VgR-mediated Vg transport system is operating in all insects, including nonsymbiotic ones (19), and that *Nasuia* is not found in all leafhoppers (18), the beginning of the *Nasuia*-associated Vg transport system, which must have been superfluous and not essential for the host at least originally, is an enigma. A possible scenario is that incorporation of Vg into *Nasuia* was beneficial for the symbiont rather than for the host at the beginning. This possibility could be experimentally tested using the current *Nephotettix-Nasuia* association. Whether *Nasuia*-allied endosymbiont lineages, such as *Zinderia* and *Vidania* (18), also incorporate Vg or not should be inspected in future studies. Finally, I point out that although Mao et al. (16) suggest that Vg-porin interaction activates the porin channel to open and allow Vg to enter the *Nasuia* cytoplasm, this claim seems dubious on the ground that porins have threshold sizes of transportable molecules, which are generally no more than 600 Da (20). Since Vg in *Nephotettix* oocytes is 178 kDa in size, the idea of porin-mediated Vg transfer into *Nasuia* cells should be critically reconsidered.

The comparative genomics of diverse insect-microbe symbiotic associations have revealed such evolutionary dynamics as recurrent degeneration, erosion, extinction, replacement, and renewal of the partnership (4, 21) as well as host-symbiont metabolic complementarity and functional fusion (3, 21). Mao et al.’s (16) finding highlights an additional layer of the complicated evolutionary dynamics regarding symbiosis as to how host and symbiont exploit mutual cellular and molecular mechanisms and establish an integrated biological system.
